# Added Breathing Resistance during Exercise Impairs Pulmonary Ventilation and Exaggerates Exercise-Induced Hypoxemia Leading to Impaired Aerobic Exercise Performance

**DOI:** 10.3390/ijerph20105757

**Published:** 2023-05-09

**Authors:** Jean-Hee Han, Min-Hyeok Jang, Dae-Hwan Kim, Jung-Hyun Kim

**Affiliations:** 1 Department of Physical Education, General Graduate School, Kyung Hee University, Yongin-si 17104, Republic of Korea; 2 Department of Sports Medicine, Kyung Hee University, Yongin-si 17104, Republic of Korea

**Keywords:** respiratory protective device, breathing resistance, exercise-induced hypoxemia, respiratory muscle load, maximal oxygen consumption

## Abstract

Protective masks impose variable breathing resistance (BR) on the wearer and may adversely affect exercise performance, yet existing literature shows inconsistent results under different types of masks and metabolic demands. The present study was undertaken to determine whether added BR impairs cardiopulmonary function and aerobic performance during exercise. Sixteen young healthy men completed a graded exercise test on a cycle ergometer under the four conditions of BR using a customized breathing resistor at no breathing resistance (CON), 18.9 (BR1), 22.2 (BR2), and 29.9 Pa (BR3). The results showed that BR significantly elevates respiratory pressure (*p* < 0.001) and impairs ventilatory response to graded exercise (reduced V_E_; *p* < 0.001) at a greater degree with an increased level of BR which caused mild to moderate exercise-induced hypoxemia (final mean SpO_2_: CON = 95.6%, BR1 = 94.4%, BR2 = 91.6%, and BR3 = 90.6%; *p* < 0.001). Especially, such a marked reduction in SpO_2_ was significantly correlated with maximal oxygen consumption at the volitional fatigue (r = 0.98, *p* < 0.001) together with exaggerated exertion and breathing discomfort (*p* < 0.001). In conclusion, added BR commonly experienced when wearing tight-fitting facemasks and/or respirators could significantly impair cardiopulmonary function and aerobic performance at a greater degree with an increasing level of BR.

## 1. Introduction

Wearing protective masks such as filtering facepiece respirators and surgical masks has become common worldwide to protect individuals from airborne contaminants and infectious agents, especially throughout SARS, MERS, and the recent COVID-19 pandemic. Further, apart from the previous recommendation, individuals who participate in physical activity and/or exercise commonly wear protective masks for airborne isolation precautions especially when physical distancing is limited [1]. However, protective masks may impose variable breathing resistances on the wearer depending on the types of masks (e.g., surgical masks, N95 vs. P100), filter properties (e.g., fiber diameter, packing layers), and fit characteristics (e.g., loose vs. tight fitting) which may adversely affect breathing comfort and cardiopulmonary function during physical activity [2]. As such, it is crucial to understand the potential impacts of protective masks on physical activity and exercise performance because this could help individuals choose the right type of masks for their exercise routines while minimizing the adverse effects of breathing resistance.

Previous studies investigating the impact of added breathing resistance due to mask wearing on physiological responses and exercise performance have provided inconsistent results, especially when tested under variable metabolic demands. For example, studies examining mask wearing during mild to moderate exercise (e.g., 2.7–5.6 km/h) showed no significant physiological impact on heart rate, breathing frequency, and oxygen saturation with slightly increased subjective discomfort, and thermal burden when tested with young, healthy individuals [3,4,5,6]. Other investigators showed that mask wearing during vigorous exercise impairs cardiopulmonary function and physical performance with increased subjective exertion as a function of breathing resistance [7,8,9,10], while others reported no significant difference in either physiological or performance variables [11,12,13].

Interestingly, two most recent studies [8,11] that examined the impact of mask wearing on physiological parameters during maximal effort exercise have demonstrated completely opposite results. Dalakoti et al. [8] tested wearing the surgical mask during maximal treadmill exercise and showed significantly reduced exercise performance (e.g., reduced exercise time, peak METs, and maximum speed) compared with the unmasked condition. On the other hand, Epstein et al. [11] tested wearing surgical masks and N95 respirator during maximal cycle exercise and indicated no differences in exercise performance between control (e.g., unmasked condition) and masked conditions, together with no meaningful changes in physiological parameters such as respiratory rate and oxygen saturation.

However, it was noted that a common limitation for drawing a definitive conclusion from previous results is either uncontrolled breathing resistance or mixed comparison between masks with different fit characteristics. For example, surgical masks are classified as protective masks, but not respirators because they do not completely seal the face, whereas a respirator such as N95 is classified as a tight-fitting mask more likely increasing breathing resistance due to the face seal [5]. Breathing resistance together with dead space microclimate are potentially major triggers for burden, especially for tight-fitting respirators [13,14]. Therefore, precise control of filter resistance and fit characteristics are of importance for better scrutinizing the potential harms of mask wearing on physiological responses and performance. 

Therefore, the present study was undertaken to determine whether added breathing resistance during graded exercise impairs aerobic exercise performance. Given the current global health crisis, the use of facemasks and other respiratory protective equipment has become increasingly prevalent, and as a result, concerns have been raised regarding the potential impact of such equipment on exercise capacity. To address this issue, three different levels of breathing resistance were tested, which are commonly applied when wearing a different grade of particulate-filtering facepiece respirators, to assess the extent to which respiratory load increased with increasing levels of resistance, and whether this led to a decreased aerobic performance and cardiopulmonary functions. It was hypothesized that respiratory load would increase in proportion to the increasing level of breathing resistance and metabolic demands, leading to reduced aerobic performance with increased subjective exertion. 

## 2. Materials and Methods

### 2.1. Participants

Sixteen apparently healthy young men volunteered to participate in the present study. The sample size was determined based on the previous results [3] that compare the pulmonary response to wearing different types of respirators using SigmaPlot software (v.12, Systat, Palo Alto, Santa Clara, CA, USA). All participants were also screened for possible risks of performing exercising and breathing under flow resistance using Physical Activity Readiness Questionnaire and spirometry tests, respectively (Table 1). Participants were excluded from the study if they reported the presence or history of smoking, respiratory diseases, cardiovascular diseases, or metabolic disorders. All participants were explained the study procedure and risks of their experimental participation before their study enrollment. Informed consent was obtained from all participants and the present study was reviewed and approved by the Institutional Review Board of Kyung Hee University (KHGIRB-21-327).

### 2.2. Experimental Design

Participants completed four trials of the experiment in a single-blind, counterbalanced manner to minimize the order effect, and all trials were separated by at least 72 h for post-exercise recovery. In order to implement breathing resistance, actual respirator filter samples from commercially available respirators were used which have been designated as Filtering Face Piece (FFP) 1, 2, and 3 according to European standards [15]. These FFP masks have been designed to protect against particulate pollutants such as contaminated aerosols and dust. FFPs with higher protection levels provide better protection, but also likely exert a greater degree of breathing resistance. 

**Figure 1:**
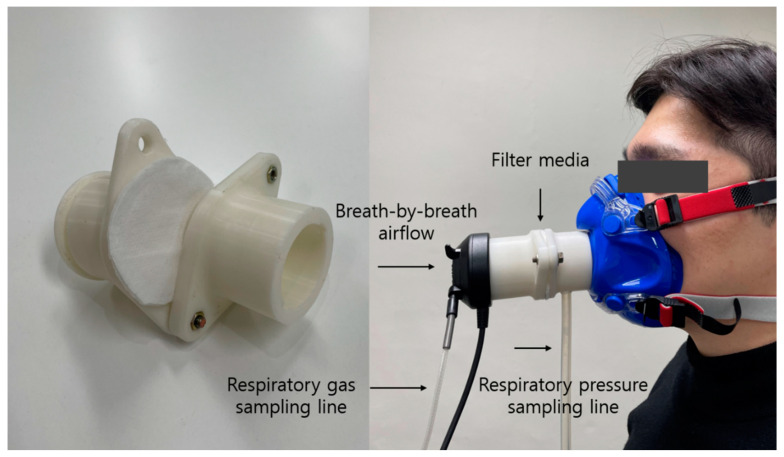
Experimental resistor and mask setup.

The breathing resistance of the three respirator samples was tested and validated at an airflow rate of 30 L/min using a breathing resistance tester (ARE-1651, ART Plus, Icheon-si, Republic of Korea). This test was triplicated with a new sample for each FFP mask, and a mean value was adopted. Therefore, four levels of experimental breathing resistance in the present study were set as no resistance (CON), FFP1 = 18.9 Pa (BR1), FFP2 = 22.2 Pa (BR2), and FFP3 = 29.9 Pa (BR3). These breathing resistances span those values permitted in the current air purifying mask guidelines by European standards [15]. Following the previously validated method [16], each level of experimental breathing resistance was tested via a custom-made breathing resistor outfitted with a metabolic mask which the participants wore during exercise (Figure 1). In essence, a plastic-based, two piece-resistor frame was made using a 3D printer and a respirator sample was implemented in between the resistor which was then connected to an oronasal facemask. In this way, the participants breathe only through the resistor while air leakage around the facial area is limited.

### 2.3. Experimental Procedure

On the days of experimental trials, participants arrived at the testing laboratory at the same time in the morning or afternoon. They wore T-shirts, shorts, and athletic shoes for testing. Then, they were equipped with the aforementioned metabolic masks with a resistor; however, experimental resistance was blinded until the completion of all trials.

For the study measurements, expired air samples were collected breath by breath and analyzed using a metabolic cart (Quark CPET, COSMED, Rome, Italia) for oxygen uptake (VO_2_), respiratory exchange ratio (RER), minute ventilation (V_E_), and respiratory rate (RR). All respiratory gas data were averaged as 1 min values for later analysis. The breathing resistor was also equipped with a digital barometric pressure sensor (PTB210, Vaisala Co., Helsinki, Finland) at the point between the mouth and a resistor medium to measure changes in respiratory pressure which could indirectly show respiratory muscle load (Figure 1). Respiratory pressure was demonstrated as positive and negative respiratory pressure during inhalation and exhalation, respectively. Heart rate (HR) and oxygen saturation (SpO_2_) were measured with a chest-worn heart rate monitor (BioHarness, Zephyr Technology, Annapolis, MD, USA) and a pulse-derived oximetry test (Oxy-Go, Roslyn, WA, USA), respectively. Ratings of subjective exertion (RPE) and breathing discomfort (BD) were measured using the Borg Ratings of Perceived Exertion and previously established 7-point breathing-effort scale (1: no discomfort—7: intolerable discomfort) [17], respectively.

Following the experimental instrumentation, participants sat and rested on a cycle ergometer (Aerobike-2, Combi Co., Tokyo, Japan) for 10 min after which baseline measurements were attained, and then they performed a graded exercise test. The exercise protocol was modified from the previous study [16] and consisted of starting at 30 Watts with an exercise load increase of 30 Watts every 2 min until 200 Watts, and thereafter an exercise intensity increase of 15 Watts every minute until volitional fatigue. During the exercise, participants were asked to maintain a pedaling rate at a minimum of 50 rpm and verbally encouraged to bring about their maximal effort. When volitional fatigue was reached, all final measurements were taken, and the participants commenced a cool down at 30 Watts of intensity for 5 min.

### 2.4. Statistics

The data analysis for the present study was conducted using SPSS (v.25 SPSS Inc, Chicago, IL, USA). To perform the analyses, two-way repeated measures ANOVA was utilized (breathing resistance levels x exercise intensity). The Greenhouse-Geisser correction was applied to the analyses to account for potential violations of sphericity. In cases where a significant interaction was observed, post-hoc pairwise comparisons with Bonferroni adjustment were conducted to explore the effects of breathing resistance on physiological and subjective variables. Additionally, one-way ANOVA was used to compare single-measure outcomes, such as participants’ maximal power output and endurance time. The statistical significance was set at *p* < 0.05, and all data were presented as the mean and standard deviation.

## 3. Results

### 3.1. Exercise Performance

**Figure 2:**
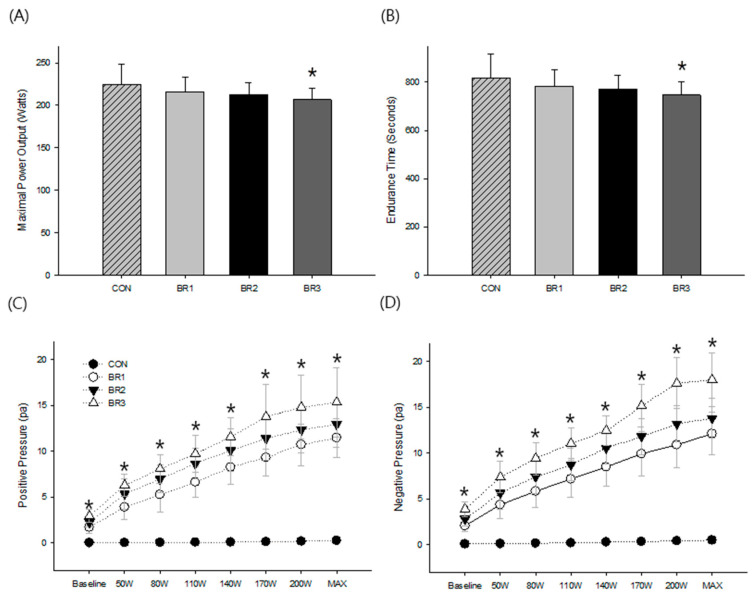
The summary of (**A**) maximal power output, (**B**) exercise endurance time, (**C**) Positive respiratory pressure (during exhalation), and (**D**) Negative respiratory pressure (during inhalationexhalation). Values are means and standard deviations * Significant difference compared to CON (*p* < 0.05).

During the study, all participants successfully completed the exercise at a minimum of 200 Watts in all experimental conditions, indicating that they were able to maintain a high level of physical performance regardless of level of breathing resistance. Both maximal power output and endurance time tended to decrease as the level of breathing resistance increased; however, a significant difference was found only between CON and BR3 (F = 2.80, *p* < 0.05) (Figure 2A,B), suggesting that while the reduced exercise performance associated with increased breathing resistance may have an overall negative impact on exercise performance, this effect may not become significant until a certain threshold is reached.

### 3.2. Respiratory Pressure

The present results indicated a significant interaction between breathing resistance and exercise intensity for both positive (F = 80.35, *p* < 0.001) and negative (F = 53.37, *p* < 0.001) respiratory pressure. These respiratory pressure changes were immediately present during the baseline, such increments became greater as exercise intensity increased indicating that the respiratory system was actively responding to the demands of exercise, while no noticeable changes in respiratory pressure were observed in CON during exercise (Figure 2C,D). 

### 3.3. Cardiopulmonary Response 

**Figure 3:**
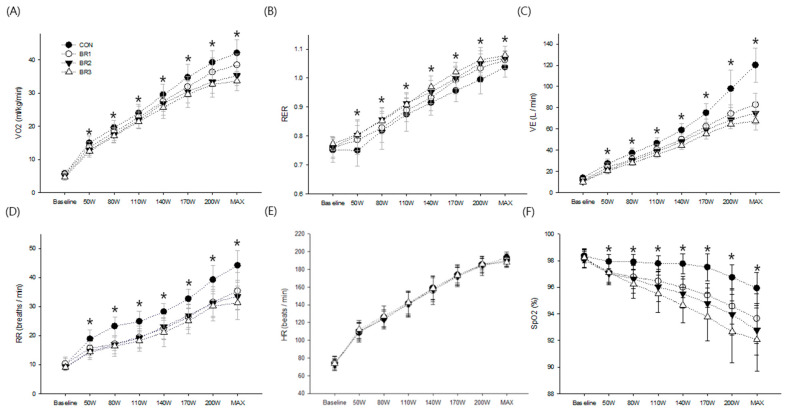
The summary of (**A**) oxygen uptake, (**B**) respiratory exchange ratio, (**C**) minute ventilation, (**D**) respiratory rate, (**E**) heart rate, and (**F**) oxygen saturation. Values are means and standard deviations * Significant difference compared to CON (*p* < 0.05).

There was a significant interaction in all measured cardiopulmonary variables between breathing resistance and exercise intensity except HR (Figure 3). The increases in VO_2_ (F = 11.74, *p* = 0.05), V_E_ (F = 42.98, *p* < 0.001), and RR (F = 10.45, *p* < 0.001) at a given exercise intensity were significantly diminished with an increased breathing resistance. Especially the observed reductions in VO_2_ and V_E_ with increased breathing resistance may be indicative of decreased gas exchanged efficiency due to breathing resistance. Further, SpO_2_ significantly decreased (F = 14.63, *p* < 0.001) as exercise intensity increased at a greater degree with a higher breathing resistance. RER also demonstrated a significant interaction (F = 1.79, *p* < 0.05) in that RER increased in all breathing resistance conditions compared to CON, but no significant differences were found among breathing resistance conditions.

### 3.4. Subjective Measures

**Figure 4:**
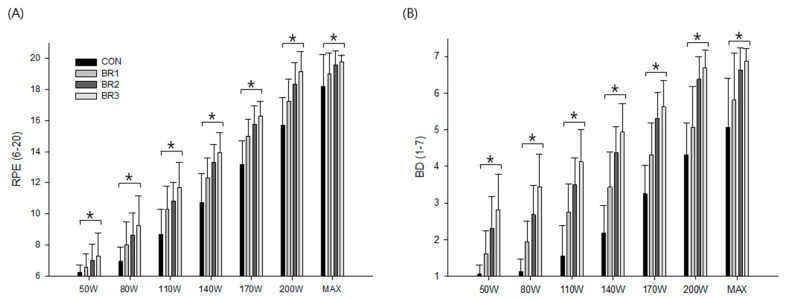
The summary of (**A**) rating of perceived exertion and (**B**) breathing discomfort. Values are means and standard deviations. * Significant difference compared to CON (*p* < 0.05).

The presented results indicated a significant interaction in RPE (F = 5.8, *p* < 0.001) and BD (F = 11.3, *p* < 0.001) between breathing resistance and exercise intensity (Figure 4). Specifically, RPE and BD were shown to be greater at each exercise intensity in breathing resistance conditions compared to CON; however, no significant difference was noted between BR 2 and 3, suggesting that while higher levels of breathing resistance may lead to greater overall exertion and discomfort, the magnitude of this effect may reach a plateau at a certain level of breathing resistance.

## 4. Discussion

This study aimed to investigate the potential effects of external breathing resistance on exercise performance and cardiopulmonary responses; therefore, three different levels of breathing resistance were tested during a graded cycling exercise. The present findings demonstrated that added breathing resistance, commonly experienced when wearing protective facemasks and/or respirators [15], could significantly impair aerobic exercise performance and exaggerate subjective exertion. Based on the present measures, the main physiological burden adversely affecting physical performance was suppressed elevation in V_E_ to an increasing level of metabolic demand together with elevated respiratory pressure which in turn led to a significant reduction in VO_2_ and SpO_2_. These cardiopulmonary impairments were observed to be greater in proportion to an increasing level of breathing resistance supporting our hypothesis.

The most intriguing observation was that breathing resistance causes a marked degree of hypoventilation linearly with increased breathing resistance at a given metabolic demand throughout the mild, moderate, and vigorous exercise stages supporting previous findings [8,9,10]. Conversely, others reported little or no significant changes in exercising V_E_ [2,17]. However, they employed continuous exercise at very low to moderate intensity (e.g., walking at 2.7~5.6 km) during which the required increase in V_E_ to meet the demands of exercise is quite low and therefore probably not significantly challenged by breathing resistance as previously assessed [12]. Further, consistent with previous results [16,17,18,19], suppressed V_E_ compared to CON at each breathing resistance becomes greater as exercise intensity increases as shown in V_E_ reduction by 14.5–31.1% in BR1, 23.1–37.6% in BR2, and 27.5–43.8% in BR3 (baseline–exercise cessation compared to CON). This incremental reduction in V_E_ was most likely caused by a lower breathing rate and reduced tidal volume due to airway resistance [20] which would lead to a greater deprivation at a higher ventilatory demand. 

Moreover, the suppressed V_E_ was associated with a progressive reduction in SpO_2_ ranging from 4.5 to 6.1% at the cessation of exercise. Presently observed mild to moderate exercise-induced hypoxemia [21,22], with breathing resistance and its consequence on VO_2max_ and exercise performance, was likely due to a reduced alveolar ventilatory diffusion [23,24]. Especially, the significant reduction in VO_2max_ at the volitional fatigue was highly correlated with SpO_2_ (r = 0.98, *p* < 0.001) in all conditions while the performance variables (e.g., the maximal power output and endurance time) significantly differed only between CON and BR3. As expected, such breathing resistance-induced reduction in SpO_2_ did not appear during low- to moderate-intensity exercise [3,4,5] while elevated CO_2_ retention and/or end-tidal CO_2_ were frequently noted [3,4,5,10]. Interestingly, two previous studies that utilized strenuous exercise while wearing facemasks reported no changes in SpO_2_ [11,25]. However, significant reductions in SpO_2_ coinciding with reduced VO_2max_ were also observed in other studies [9,16] that controlled mask sealing for air leakage during exercise, similar to the present study, implicating that facemask fit affects airflow limitation and could variably influence cardiopulmonary function [3,4,17]. Further, the most recent study that investigated the impact of mask wearing (e.g., surgical mask and N95 respirator) during maximal cycle exercise, similar to the present study, showed no significant impact on RR while V_E_ and VO_2_ data are not available. We believe these results are mostly due to uncontrolled air leakage during mask wearing, thereby influencing the pulmonary variable when considering breathing through a resistance medium likely causes slow breathing to be demonstrated due to suppressed RR [2,5].

Some of the present results were somewhat expected when considering a degree of flow resistance is determined by material resistance (e.g., mask filter) and airflow rate (e.g., V_E_) [13] as the former is constant at each condition and the latter increases during graded exercise in the present setting. Therefore, a level of breathing resistance would increase when V_E_ increases linearly with increased metabolic demand. This phenomenon was also confirmed by a linear increased in respiratory pressure in both inhalation and exhalation during incremental exercise in a similar fashion to the aforementioned V_E_ responses, which indirectly shows the elevated work of breathing adversely affects physical performance, especially during vigorous intensity exercise [18,22]. The presented demonstrated changes at each breathing resistance (Figure 2C,D) particularly indicate an increase in the non-elastic component in breath work to overcome flow resistance [23]. This flow limitation has been shown to induce diaphragmatic fatigue [23] which consequently alters sympathetic outflow and limb blood flow, resulting in premature fatigue and impaired physical performance [26,27]. While the presented results clearly showed a hierarchical elevation in respiratory pressure as a function of breathing resistance, further investigations are needed to determine the metabolic costs of breath work when wearing a mask during dynamic exercise.

In addition, some previous studies [3,5,13] have shown that breathing difficulties may even occur with very low-resistance filtering masks, such as N95, while another study [17] reported only minimal or clinically insignificant perceptual differences in breathing effort and discomfort during low to moderate exercise. However, the present results indicated significantly deteriorated perceptual responses in both RPE and BD. Further, such impaired perceptions were positively related to a level of added breathing resistance in agreement with previous findings [2,3,7,9,18,19] and likely adversely affected exercise tolerance shown as a significantly elevated perceived exertion and breathing discomfort at all stages of exercise. 

Some of the present results must be interpreted with caution, as breathing resistance in this study was implemented via airflow leakage-controlled resistor, but not in the form of mask wearing commonly utilized in other studies [3,6,7,8,9,10,11,12,13,14] and experienced in daily life while the resistance materials were taken from commercially available respirators. However, it should also be noted that FFP-class masks in Europe and/or others in the U.S. (e.g., National Institute for Occupational Safety & Health approved N, R, P class filtering facepiece respirators) are classified as tight-fitting masks that, if worn correctly, with minimum facial leakage during activity are believed to exert a similar degree of breathing resistance in this study. 

Further, as shown in the present results, breathing resistance (e.g., measured by respiratory pressure) changes depending on V_E_ during incremental exercise while a mask and/or respirator during the approval process is tested at a constant flow rate; for example, 30, 95, and 160 L/min for European FFP masks [15] and 85 L/min for NIOSH approved respirators [28]. Therefore, the design and performance characteristics of masks and/or respirators need to be carefully considered to better examine the impact of mask-derived breathing resistance on physiological strain and as a cause of performance impairment.

Some limitations should be noted. First, as mentioned above, breathing resistance was implemented using a custom-made resistor; thereby, actual airflow resistance when wearing a filtering facepiece respirator may appear differently. Second, we recruited only young healthy men which may influence the external validity of these findings for other populations. Lastly, despite being large enough to detect significant differences in the main outcomes, the small sample size of sixteen subjects may limit the explanation for small changes in secondary outcomes, such as RR and HR between different BR conditions.

## 5. Conclusions

In conclusion, breathing resistance that is commonly experienced when wearing tight-fitting facemasks/respirators could significantly impair ventilatory responses to moderate and vigorous exercise, consequently causing mild to moderate exercise-induced hypoxemia, leading to a decrement in aerobic performance. These findings highlight the importance of carefully considering the potential impact of respiratory protective equipment (e.g., masks, respirators) on exercise capacity, particularly in individuals who engage in regular physical activity or perform physically demanding work. Future studies are warranted to investigate threshold resistance entailing cardiopulmonary impairment during dynamic exercise together with the assessment of additional metabolic burdens for breath work under added breathing resistance. By addressing these issues, future investigations could provide a more comprehensive understanding of the impact of breathing resistances caused by respiratory protective equipment on associated decrements in cardiopulmonary functions and exercise performance.

## Figures and Tables

**Table 1:** Participant characteristics (*n* = 16).

Variable	Mean ± SD
Age (yrs)	22.3 ± 3.1
Height (cm)	176.4 ± 4.8
Weight (kg)	75.3 ± 8.3
Systolic blood pressure (mm Hg)	120.1 ± 5.0
Diastolic blood pressure (mm Hg)	71.5 ± 5.1
Forced vital capacity (FVC; L)	5.3 ± 0.6
Forced expiratory volume in one second (FEV1; L)	4.3 ± 0.1
FEV1/FVC (%)	0.8 ± 0.1
Maximal voluntary ventilation (L)	175.9 ± 21.3

## Data Availability

Not applicable.
